# Effectiveness of Adaptation of a Resilience-Building Intervention Among Individuals With Adverse Childhood Experience: Protocol for a Randomized Controlled Trial

**DOI:** 10.2196/56826

**Published:** 2025-02-12

**Authors:** Jun Kiat, Mahadir Ahmad, Caryn Mei Hsien Chan, Satirah Zainalabidin, Michael Ungar, Ponnusamy Subramaniam

**Affiliations:** 1 Clinical Psychology and Behavioural Health Program National University of Malaysia Kuala Lumpur Malaysia; 2 Programme of Biomedical Science, Centre of Toxicology and Health Risk Study (CORE) Faculty of Health Sciences, Universiti Kebangsaan Malaysia Kuala Lumpur Malaysia; 3 Cardiovascular and Pulmonary (CardioResp) Research Group Universiti Kebangsaan Malaysia Bangi, Selangor Malaysia; 4 Resilience Research Centre Halifax, AB Canada

**Keywords:** adverse childhood experience, resilience, resilience-building intervention, young adults, stress, psychological well-being

## Abstract

**Background:**

The impact of adverse childhood experiences (ACEs) has been the focus of most studies for the past decade. There is an indication that developing resilience can help youth overcome these ACEs.

**Objective:**

This article presents a study protocol for a randomized controlled trial (RCT) to investigate the effects of a resilience-building intervention on psychological well-being, coping strategies, stress, quality of life, resilience, resource finding, and resilience among individuals affected with ACEs in Malaysia.

**Methods:**

The is a 2-armed, single-blind, RCT, whereby 50 participants (25 in each group) with ACEs will be randomly assigned to intervention and control groups. The former will be exposed to a resilience-building program (R2), which entails a multisystemic approach to resilience and recognizes the importance of rugged qualities and access to resources among individuals affected with ACEs. The intervention will be delivered via internet-based by a facilitator and broadly divided into 5 sessions, focusing on self-exploration and social support, coping techniques and coping skills, resource finding, spirituality, and resilience building. Meanwhile, the control group participants will not receive any form of intervention. Saliva samples will also be collected from both groups and assessed for salivary cortisol levels. Outcome measures will be assessed during baseline and postintervention using validated instruments. Another follow-up measurement will be conducted 4 weeks later.

**Results:**

The clinical trial has been registered with the Australia New Zealand Clinical Trials Registry. Ethical approval was obtained from the Research Ethics Board at the National University of Malaysia (UKM PPI/111/8/JEP-2021-894). A total of 28 participants have been recruited to the RCT Participant recruitment will be completed by January 2025. The final analysis will be conducted by March 2025.

**Conclusions:**

This is among the first studies to provide evidence in the context of RCTs for resilience-building intervention that combines self-report and physiological measures (ie, saliva and heart blood pressure) among individuals with ACEs. The findings will assist relevant authorities in the health and policy sectors to develop effective strategies for addressing the negative impacts of ACEs on the vulnerable population in Malaysia.

**Trial Registration:**

ACTRN12622000604707; https://www.anzctr.org.au/Trial/Result/DataSharingStatement.aspx?id=383614

**International Registered Report Identifier (IRRID):**

DERR1-10.2196/56826

## Introduction

### Background

Adverse childhood experiences (ACEs) are stressful or traumatic events faced by individuals that could have a pervasive impact throughout the developmental stages of their lives through psychological and physiological mechanisms, particularly when the consequences are neglected [1]. ACE can have profound effects on individuals of all ages [2,3]. These experiences may culminate in disrupted development of brain areas that are stress-sensitive [4,5], which are reflected in behavioral alterations.

For example, individuals with ACEs may face challenges with emotion regulation, thereby leading to diverse negative consequences such as difficulties with interpersonal relationships, alcohol, and smoking habits [6,7]. Overall, these events are associated with obesity, depression, suicide, anxiety, heart attack, chronic health problems, chronic obstructive pulmonary disease, asthma, stroke, cancer, and unemployment [8,9]. Apart from emotion regulation, ACE survivors’ experience deficits in several domains of executive functions [10], which impact their well-being and psychological health [11]. Evidence suggests that individuals with ACEs, particularly children and young adults, are disadvantaged in terms of coping with increased stress relative to their peers without such bad experiences [12]. Since young adults are still experiencing biological and psychosocial development, the consequential impacts of ACE can be profound during adolescence and young adulthood [12].

Mental health problems are common among children and adults, which are predominantly presented as depression or anxiety and are characterized by frequent comorbidities [13]. Studies have demonstrated that ACEs represent an important predictor of emotional problems [14]. Previous research has documented several underlying events for the association between emotional problems and early ACEs among young children and adults [13,14]. For instance, individuals with a history of early ACEs were more accurate in recognizing threatening stimuli [15], constituting an adaptive response in harmful conditions. Nevertheless, it can heighten the risk of mental health disorders over time [16]. While individuals with ACEs are more likely to adopt maladaptive emotional regulation skills such as disengagement, expressive suppression, and rumination, they are less likely to perform successful activities such as cognitive appraisal [15], thereby leading to early emotional anomalies [16].

Specifically, ACEs also constitute a significant barrier to an individual’s capacity for resilience. Resilience depicts the resources, capability, and processes available to a system or an individual to adapt successfully in the face of challenges or adversities [17-19]. Resilience encompasses the factors that facilitate positive adaptation and navigation toward resources to sustain well-being in the context of adversity [16]. Therefore, researchers have developed an interest in resilience intervention as a potential approach to addressing the negative impacts of ACEs [19]. For instance, cognitive behavioral therapy (CBT) has been used in resilience interventions in ameliorating health problems, particularly among adults with a history of ACEs [19-21]. Hence, building resilience in individuals with adversity in the context of psychological intervention offers a new approach to managing individuals with ACEs.

### Adverse Childhood Experiences and Negative Consequences Among Children and Adults in Malaysia

ACEs refer to neglect, abuse, and dysfunctional households that may be deleterious to a person’s health and well-being. Research conducted in Malaysia has depicted an increasing frequency of child abuse and neglect cases, which are typical examples of ACEs that may impact negatively on physical and mental health [22]. ACEs also contribute to the growing health care expenditure, as the health consequences of ACEs reportedly accounted for 2% of the gross domestic product [23].

Nevertheless, information on the prevalence and negative impacts of ACEs among children and adults in Asia, specifically Malaysia, is scarce due to limited research. A recent study revealed a high prevalence of various ACEs among university students in northeast Malaysia, with a report of emotional abuse, emotional neglect, physical abuse, and sexual abuse occurring in 30.2%, 29.2%, 28.7% and 9.1% of the studied population [22]. High-risk behaviors (HRBs), particularly physical inactivity and community violence were recorded among 39.3% and 54.5% of the students, respectively [22].

Apart from HRBs, depression has been identified as a consistent outcome of ACEs among young adults in Malaysia [24,25]. Young individuals from Shah Alam, Malaysia, exhibited a high level of depression and ostracism, which was associated with ACEs. Early psychological mistreatment such as parental neglect of a child’s needs can lead to poor mental well-being [25], externalizing and internalizing problems, as well as anxious and avoidant attachment styles, making it challenging for children to develop a sense of belonging and feeling ostracized [26,27]. Therefore, early life traumatic events and ignorance appear to have strong influences on psychological well-being among this vulnerable population.

Besides the younger population, older adults in Malaysia have also been shown to be affected by ACEs, which may ultimately transit into elder abuse [28]. Furthermore, the risk of elder abuse increased with the cumulative number of ACEs. The results depict how early life adversities play an important role in older adults’ victimization. Recognizing the possibility that vulnerability to maltreatment can persist throughout the life course of elderly individuals is critical when attempting to address the problem through emotional and social support.

### Resilience-Building Interventions for Individuals With Adverse Childhood Experiences

Resilience is considered to be pertinent in the association between emotional problems and ACEs. Studies have depicted that resilience is a protective factor that can propel an individual to successfully address adverse experiences [29]. Thus, resilience is regarded as playing a protective role in the relationship between emotional disorders and ACE [29-31].

ACEs constitute a public health crisis that requires a wide range of interventions due to their high prevalence and impact on health disparities [31,32]. Resilience interventions are typical examples of CBT that are effective in ameliorating mental health disorders faced by individuals with ACEs [15]. According to Iniguez and Stankowski [33], ACE research has yielded strong evidence to support claims that “resilience resources and well-timed interventions to promote resilience can ameliorate the negative effects of ACEs” [33].

Chandler et al [21] demonstrated the feasibility and efficacy of an empower resilience intervention to enhance resilience and health behaviors among young adults with a history of ACEs. By using a 2-arm pre-post repeated measures design, young adults in the intervention group recorded significantly higher scores for building strengths, creating support connections, and reframing resilience. Meanwhile, a face self-help app designed by Brodbeck et al [34] and based on cognitive-behavioral principles, is currently being tested for its efficacy in promoting resilience and well-being in emerging adults with a history of ACE. MacIsaac et al [35] also evaluated the effects of an innovative, smartphone app-based resilience intervention on first-year university students’ self-regulatory skills (ie, emotion regulation and executive functioning), and the mediating role of emotion regulation. After 4 weeks of using the app, students’ emotion regulation and depressive symptoms improved significantly, with a faster rate of change in emotion regulation among those with more ACEs. Thus, evidence suggests that app-based resilience intervention can assist young adults with ACE by improving their emotion regulation skills and mitigating depression.

Systematic review findings depict that most interventions for addressing ACEs focused on psychological interventions and mental health outcomes, and cognitive-behavioral therapy has been consistently found to be effective in mitigating the negative impacts of abuse [36]. On the other hand, findings from interventions involving psychological therapies, specific support such as parent training, and broad support interventions are generally inconclusive despite some promising results [36]. In summary, significant gaps exist in the available evidence on interventions for ACEs, with most research focusing on individual psychological effects while neglecting the social pathways which may indirectly influence the negative impacts of ACEs. Several areas such as social relationships, life circumstances and health behaviors are examples of several negative impacts of ACEs that are still under investigation in most intervention research [34-36].

Building upon the global view on ACE research and resilience-building interventions, the Malaysian context must be taken into account in order to develop an effective intervention to address the problem of ACEs among children and young adults. Literature findings from the studies conducted in Malaysia have shown that ACE is a multifactorial problem that requires a multidimensional approach, rather than focusing on one individual aspect [22,23]. Such an approach is well-described in the multisystemic model of resilience developed by Ungar [8], highlighting the capacity of biopsychosocial and social-ecological systems to support external and internal conditions for well-being, as well as improving diverse populations’ quality of life. Thus, resilience encompasses the process whereby individuals harness the resources that are necessary for them to function optimally and seek resources to be provided using meaningful approaches, either contextually or culturally [8].

Interventions that emphasize individual change without considering the environmental domain may yield short-term benefits [37]. Hence, intervention should have a dual focus, encompassing personal and environmental change. For instance, a child’s resilience is a product of both their capacity to cope effectively under stress and the capacity of their physical and social environments to facilitate positive development. Under conditions of normal stress, individual ruggedness may be sufficient to support well-being; nevertheless, resources become more important and required as the individual experiences greater barriers to functioning. These 2 broad aspects form the foundation of the multisystemic perspective of resilience “R2,” which was coined to affirm the need to mitigate both the rugged qualities of individuals and their access to resources) [38].

Research has shown that individuals with more internal capacities such as problem-solving, self-regulation, and positive future orientation are more likely to harness opportunities for relationships and exploit such opportunities for academic or financial success [39,40]. Likewise, higher levels of motivation to execute life tasks and being more optimistic were observed among individuals with better access to external resources [41,42]. These findings reflect the dynamicity of resilience, whereby individuals interact with the world around them to make the best use of available resources despite exposure to adversity or atypical stress.

As highlighted in the reviewed resilience-building interventions for ACEs, while some target the public and common adversities like job burnout [43] and stress [44], others are aimed at specific groups, such as chronically diseased individuals [45], employees returning to work [46], and health care workers [47]. These interventions are delivered using diverse methods, ranging from phone apps to web-based tools or printed manuals, and duration may be as short as single-day workshops to weekly or monthly sessions. However, a recent meta-analysis revealed that such interventions have a small to moderate effect on improving resilience [44,47]. Existing resilience interventions also differ in terms of the targeted protective factors, such as self-efficacy, problem-solving skills, and cognitive flexibility, with most focusing on rugged factors. Only a few interventions consider resources that are external to individuals with ACEs [48]. Accumulated evidence suggests little to no data on interventions that explicitly target both coping strategies and ways of creating better-resourced environments around individuals with ACEs.

Hence, this study will modify and implement an R2 resilience program based on a resilience-building intervention designed with the principles of implementation science [38]. The multisystem aspect of the intervention allows it to focus on multiple systems for the individual’s change process and adaptation process around life circumstances. It is a curriculum-based approach that integrates all the well-researched factors and presents equal emphasis on the surrounding environments to the individual [8,38]. Thereafter, the factors and aspects can be modified and implemented into the resilience-building intervention accordingly. This study aims to explore the effects of the resilience-building intervention on mental health, stress, resilience, and resource finding among individuals affected with ACEs in Malaysia.

## Methods

### Study Design and Setting

This is a 2-armed, single-blinded, randomized controlled trial (RCT) that will be conducted among Malaysian youths with ACEs at the Department of Clinical and Health Psychology, National University of Malaysia. Briefly, the intervention group includes an evidence-based curriculum and components such as emotion regulation, active coping and goal setting, cognitive flexibility, mindfulness-based stress reduction, social support, self-exploration, resources and navigation, and finally, spirituality and religion. Participants in the control group will not receive any form of intervention. The main outcome of this study is resilience among youths with ACE while the secondary outcomes include coping, psychological well-being, quality of life, subjective stress, perceived stress, personal resources, and adult resilience. These variables will be evaluated at baseline and postintervention. Intention-to-treat analysis will also be performed. This clinical trial has been registered with the Australia New Zealand Clinical Trials Registry (trial Id: ACTRN12622000604707).

#### Sample Size

Sample size calculation was performed using G*power 3.1 software (Heinrich-Heine-Universität Düsseldorf). By assuming a study power of 0.8, 95% CI (α=0.05), and a moderate effect size of 0.25 as recommended by Ferguson (2009), the *F*-test was selected alongside repeated measures ANOVA with in-between interaction, with 2 groups and the number of measurements. Thus, a total sample size of 36 was obtained, reflecting 18 participants per group. We considered a high drop-out rate of 50% as several individuals may be unwilling to participate given the adverse effects of ACEs and confidentiality in sharing experiences with the researchers. Therefore, the sample size was increased to 50, amounting to 25 participants per group.

#### Eligibility Criteria

The inclusion criteria entail participants aged 18 to 30 years old and an individual who scores more than 4 or higher levels of ACE. This age group spans the young adulthood phase of an individual, and it is considered a significant developmental stage characterized by exposure to unique challenges and opportunities [21]. Meanwhile, the exclusion criteria are individuals receiving any type of intervention, demonstrating any severe psychopathology or psychiatric illness that requires a psychopharmacological approach, and those who had received intervention or therapy consistently in the past. Individuals need to fulfil the criteria to be recruited as mentioned above. Individuals will be recruited through multiple centers and social media platforms

#### Participant Recruitment and Randomization

The participants for this study are Malaysian youths with ACE, particularly those visiting the Psychological Department at the National University of Malaysia Medical Centre. During the selection stage, participants will be approached by the researcher and seek their consent to participate in the study. Those providing affirmative responses will then be instructed to fill up the self-administered ACE questionnaire.

This assessment period is the screening stage to identify those that fulfil the inclusion criteria. Participants who score four or more of ACE will then be included in a pool of participants and assigned randomly to either the control group or intervention group. Random assignment procedure will be implemented by random allocation software, Research Randomiser [31], to generate numbers and assign participants to an intervention group or control group. Allocation concealment will be performed by a single blinding procedure, whereby the assessors of the outcome measures will be unaware of the specific group the participants belong to. In other words, only the investigator allocating participants to either the control or intervention group will be aware of this information while other assessors are blinded. Table 1 depicts the participant enrolment schedule, time points and assessments to be performed during the study period.

**Table 1:** SPIRIT (Standard Protocol Items: Recommendations for Interventional Trials) schedule of enrolment, interventions, and assessments.

			Study period							Close-up
			Enrolment		Allocation		Postintervention		Four-week follow-up	
Timepoint			t_1_		0		t_2_		t3+4weeks	
**Enrolment**			✓							
	Screening	✓								
	Eligibility screen	✓								
	Informed consent	✓								
	Allocation			✓						
**Interventions**										
	Intervention A									
	Control									
**Assessments**										
	Brief Coping Orientation to Problem Experienced Scale - English and Malay version	✓				✓		✓		
	Ryff’s Scale of Psychological Well-being (SPWB) - English and Malay version	✓				✓		✓		
	Quality of Life –WHOQL-BREF - English and Malay Version	✓				✓		✓		
	Subjective stress – Perceived Stress scale - English & Malay version	✓				✓		✓		
	Personal Resources Questionnaire 2000 - English and Malay version	✓				✓		✓		
	Adult Resilience Measure-Revised - English and Malay version	✓				✓		✓		
	Biomarker - measure changes in salivary cortisol	✓				✓				
	Blood pressure (blood pressure measured with a sphygmomanometer	✓				✓				

**Figure 1:**
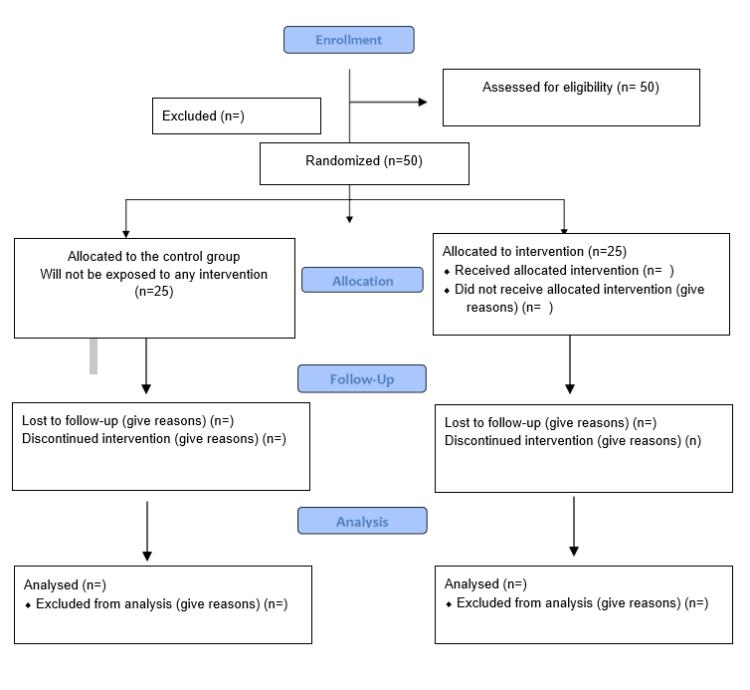
Consolidated Standards of Reporting Trails 2010 flow diagram.

Participants in the control group will receive no intervention, while those in the intervention group will receive five sessions of resilience-building intervention conducted by a trained facilitator. Hotlines, post session, and appropriate resources will be provided for those reporting distress after the intervention. Investigators will be blinded throughout the whole process of randomization and data collection as the whole process is handled by a research assistant (randomization), counsellor, and clinical psychologist (data collection). Under no circumstances will unbundling occur as a research assistant, counsellor, and clinical psychologist are briefed with the standard operating procedure to follow when an incident occurs. The flow diagram is presented in Figure 1.

#### Contents and Delivery Method of the Intervention

The intervention will be adapted and modified according to the R2 Resilience building program designed by Ungar [20], which comprises 7 principles; help people to navigate, help people to negotiate, think about systems, coordinate services and supports, provide continuous support, be relevant to place and culture, and share responsibility for solutions. These principles will guide the process of modifying the intervention and the principles will be reflected in each session. The intervention will be delivered in 5 sessions.

Session 1 is divided into subsessions entitled “Self-exploration” and “Social support.” The first subsession focuses mainly on building rapport with the participants and helping them explore their coping skills, strengths, weaknesses, and talents. The second subsession emphasizes the importance of social support to enhance a sense of belonging and connection, as well as explore resources relating to social support. Both sessions will be delivered via internet-based with each lasting for 60 minutes.

The second session is also divided into 2 parts, coping technique including cognitive flexibility and grounding technique, and coping skills. The coping technique focuses on participants’ coping strategies and a deeper insight compared with the first session. This section also aims to provide cognitive exercise for preventing catastrophic thinking. As for coping skills, the strategies will be discussed in depth and practiced together with the facilitators. Participants will also be directed to work in pairs and assisted to identify their best coping strategies, either emotion-focused or solution-focused.

The third session comprises 2 parts to familiarize the participant with resource findings and explore their understanding of resilience. First, the facilitator will recap what the participant has learnt in the previous sessions and help identify where they can get help and access the available resources. Such assistance will be tailored for each participant to learn about resilience resources and harnessing them accordingly. In the second part, in-depth discussion will be held with each participant by focusing on past ACEs and how they were able to overcome the experiences, as well as their past resilience resources. Both sessions will be delivered via internet-based with each lasting for 60 minutes.

The fourth session is also divided into 2 subsessions; (1) what is resilience to you (part II), and (2) spirituality. The first subsession is the continuation of the second part of the third session with the same components as shown in Table 2. Meanwhile, the second subsession entails learning from each other regarding the role of religion and spirituality in addressing past adversities. The last session is the “Resilience-building (wrap-up),” whereby all the intervention components will be reviewed and visualized in the form of a diagram. Each session of the intervention will last for 2 hours (60 minutes for each session, with 15-minute breaks between sessions). Each session will be conducted weekly, week 1 for the first session and week 5 for the fifth session respectively. All sessions of the intervention will be administered via internet-based to each participant by the facilitators.

**Table 2:** Components of the intervention.

Week	Session	Session	Duration	Mode of delivery	Focus
Week 1	1st session (1A)	Session 1A: Self-exploration	60 minutes	Internet-based	Build rapport among students with an ice-breaking activity To help participants explore their coping skills, strengths, weaknesses, and talents
Week 1	1st session (1B)	Session 1B: social support	60 minutes	Internet-based	To talk about the importance of social support To foster a sense of belonging and connection. To explore resources regarding social support
Week 2	2nd session (2A)	Session 2A: coping techniques: cognitive flexibility and grounding technique	60 minutes	Internet-based	Explore participants’ coping strategies (be mindful of labelling participants’ strategies). Go deeper from session 1A Cognitive exercise to prevent catastrophic thinking How do you practice cognitive exercise? What is fallacy thinking? Introduce A-B-C and how to practice them
Week 2	2nd session (2B)	Session 2B: coping skills	60 minutes	Internet-based	Discuss the strategies in depth and practice them together. (Follow up from Session 2A) Finding allies (participants form a pair and check in with each other) Emotion-focused coping vs. solution-focused coping During a crisis, how do these routines help? Helping the body to feel safe
Week 3	3rd session (3A)	Session 3A: resource finding	60 minutes	Internet-based	Review and monitor from the previous session to recap what has been learned To help participants know where to get help and the resources available To help participants learn about their resilience resources and how to tap into them
Week 3	3rd session (3B)	Session 3B: what is resilience to you?	60 minutes	Internet-based	How do you survive? What is the meaning of your stories? What are your resilience resources in the past?
Week 4	4th session (4A)	Session 4A: what is resilience to you? part II	60 minutes	Internet-based	How do you survive? What is the meaning of your stories? What are your resilience resources in the past?
Week 4	4th session (4B)	Session 4B: spirituality	60 minutes	Internet-based	Review and monitor from the previous session to recap what has been learned To learn from each other about the role of religion and spirituality
Week 5	5th session (5A)	Session 5B: eesilience-building (wrap-up)	120 minutes	Internet-based	To review and discuss all of the components To draw a diagram with all of the components involved

#### Intervention Protocol and Fidelity

The intervention will be conducted in Malay or English. It aims to encompass both languages and will be provided according to the participant’s native language. Therapists will be trained to follow the protocol and the manual. A recap will be conducted at the end of the session. Dynamic assessment will be used to track the learning progress. Participants will use journaling as a method to practice their skills. In addition, progress and practice will be discussed at the beginning of each session. As for intervention fidelity, journals and progress of group sessions will be collected from mental health professionals after each session to ensure they continue a similar structure for the intervention. The checklist will be prepared for the mental health professional.

### Data Collection Instruments and Outcome Measures

#### Adverse Childhood Experience Questionnaire

The Adverse Childhood Experience Questionnaire (ACE-Q) is a 10-item measure to assess events of traumatic or adverse experiences that an individual experienced before the age of 18 years. The instrument evaluates the individual’s exposure to childhood physical, psychological, or sexual abuse, as well as household dysfunction such as substance use, domestic violence, and incarceration. The ACE-10 has been validated among the Malaysian population with an acceptable internal consistency of 0.86 and internal validity ranging from 0.28-0.70 [49].

#### Patient Health Questionnaire-9

The English and Malay versions of the Patient Health Questionnaire-9 (PHQ-9) will be used in this study to assess depression among youths with ACEs. The instrument has been validated among the Malaysian population by Sherina et al [50] with a sensitivity of 87% (95% CI 71%-95%) and specificity of 82% (95% CI 74%-88%). It was also reportedly suitable as a case-finding instrument in Malaysian primary care clinics.

#### Brief Coping Orientation to Problem Experienced Scale

The brief coping orientation to problems experienced (COPE) instrument comprises 28 items with 14 broad coping strategies. The Malay version of the instrument was validated by Saiful [51] among secondary school adolescents and was found to be valid and reliable in identifying coping strategies. Specifically, the overall Cronbach α value was 0.83 with the majority of the coping strategies reflecting acceptable internal consistency.

#### Ryff’s Scale of Psychological Well-Being and Quality of Life

The Ryff’s Scale of Psychological Well-being (SPWB) and World Health Organization Quality of Life Assessment (WHOQL-BREF) will be used in this study to assess the psychological well-being and quality of life among adolescents with ACEs. These instruments have been validated among the Malaysian population with acceptable discriminant validity, construct validity, and test-retest reliability greater than the threshold value of 0.5 [52-54].

#### Personal Resources Questionnaire 2000

The personal resources and workability of the participants will be assessed using the Work Ability-Personal Radar (WA-PR) instrument, which has been previously translated into Malay and validated by Hamdan et al [55]. The instrument has adequate psychometric properties and has been validated with acceptable discriminant validity, construct validity, and test-retest reliability greater than the threshold value of 0.5, making it suitable to be used in investigating personal resources and workability levels in the Malaysian population [55].

#### The Perceived Stress Scale-10

The Perceived Stress Scale-10 (PSS-10) is generally used in assessing stress perception. The Malay version of the PSS-10 has been validated among diverse populations in Malaysia, with the latest being nurses from government hospitals [39]. The instrument revealed acceptable internal consistency with a Cronbach α value of 0.63 and intraclass correlation coefficient of 0.81 (95% CI 0.62-0.91) following a 7-day test-retest reliability analysis. Furthermore, previous studies found significant correlations between the stress component of Depression Anxiety and Stress Scale 21 and the total score and negative component of the PSS-10 (r=0.56-0.61). Therefore, the PSS-10 is considered valid and suitable for investigating stress perception among Malaysian youths, including adolescents [56].

#### Saliva Cortisol and Blood Pressure Assessment

Each participant will be instructed to provide approximately 2 mL of whole saliva by passive drool, which will be then split into multiple 100 uL aliquots and frozen immediately. One aliquot of each of the 50 participants’ saliva will be transported overnight on dry ice to the institutional laboratory. Cortisol analysis will be performed using commercially available immunoassay according to the manufacturer’s guidelines (Salimetrics). Meanwhile, participants’ blood pressure will be measured objectively with a sphygmomanometer.

#### Time Points for the Primary Outcomes and Retention

Premeasurements will be conducted at baseline, whereas postmeasurements will be collected after the fifth session as shown in Figure 1. Participants will be given Ringgit Malaysia 50 after the third and fifth sessions of the intervention to ensure they do not drop out of the study. Another follow-up will be conducted 4 weeks after the Intervention.

### Data Analysis

All data management and statistical analysis will be conducted using SPSS (version 26; IBM Corp). Data management will be performed by screening for missing data and checking for potential outliers. Descriptive statistics will be used to assess the data normality and to summarize the pre and postintervention scores for each group at different periods. Mean and SDs will be provided for normally distributed data, followed by mixed ANOVA with repeated measures to compare the pre and postintervention mean scores between and within the groups.

The assumptions of ANOVA include randomly collected and normally distributed data, sufficient sample size, homogeneity of variances, and absence of violation and outliers. Levene’s test will be applied to test the homogeneity of variance. Mixed ANOVA benefits RCT design as it allows testing for 2 groups across 2-time points. Mixed ANOVA analysis aims to handle response outcomes conducted on the same experimental unit at a different time or under different conditions [57]. The differential effects of the intervention components will be tested with interactions between the component and time. These analyses will model random slopes and intercepts for participants, explore the fixed effects of the condition, and test the repeated measures over time. This type of analysis is advantageous by accounting for missing values through the maximum likelihood estimation method [57]. Effect sizes will be computed based on Cohen *d*.

In addition, the intention-to-treat analysis will be applied to analyze the participants according to their assigned groups. This analysis includes all participants and ignores noncompliance, protocol deviation, and withdrawal. The intention-to-treat analysis is a complete trial strategy for the design, execution, and analysis of RCT, focusing on the consolidated standard of reporting trial guidelines. Thus, the number of participants in each assigned group will be analyzed by the intention-to-treat principle.

As for missing data, drop-out in this study refers to participants who withdraw actively from the intervention post randomization. All cases of dropouts will be considered in the intent-to-treat samples since they have been randomized and included in the analysis. The extent and pattern of missing data will be assessed, and depending on the results, missing values will be replaced by using multiple imputations. The impact of the imputation of missing values will be further explored by conducting sensitivity analyses.

#### Criteria for Discontinuing or Modifying Intervention

All the intervention modules were well understood by the participants enrolled in the pilot test; hence, no attempt has been made to review the intervention. Participants have the right to withdraw from the research at any given time if they do not feel comfortable continuing with the study. If the counselor or clinical psychologist reviews and discusses the harm of the participants continuing to participate in the research project, they will advise the participants to drop out of the study based on their professional judgment. A panel comprising counselors, and clinical psychologists will jointly review the case and make a consensus decision.

#### Data Auditing and Management

The team is required to submit first and second progressive reports to the Research Ethics committee, at the National University of Malaysia as part of the auditing process. Only group data will be reported. Data will be reported collectively; individual data will not be disclosed. Journal and progress of group sessions will be collected from mental health professionals after each session to ensure they continue a similar structure for the intervention. The checklist will be prepared for the mental health professionals.

As for data storage and management, the data will be entered digitally and stored in a Microsoft Excel sheet and Microsoft Word document by the investigator after being collected from mental health professionals. Each dataset will be assigned a code to protect the participants’ identity. The encryption of the firewall will help secure and protect the data. A passcode will be generated to protect these documents. The data will be stored in the institution for 5 years, kept confidential and used only for educational purposes.

#### Standard Operating Procedure to Manage Harm

Counsellors and clinical psychologists will evaluate if any of the participants feel discomfort or express interest in withdrawing from the studies. This assessment will be carried out during the break intervals between each session of the intervention and the follow-up periods post intervention. Participants will be asked verbally if they still feel comfortable and willing to proceed with the intervention. Follow-up and proper redirecting to resources will be practiced with individuals who dropped out of the study.

#### Protocol Amendments

Any modification to the protocol, an update will be submitted to the Australia New Zealand Clinical Trials Registry and Research Ethics Board, The National University of Malaysia, and related journal publications.

#### Recordkeeping and Specimen

Consent will be obtained from participants to collect saliva. Data obtained through this study will only be published under group data. No individual data will be identified. The saliva samples obtained will not be kept and discarded right after the cortisol level measurement and analysis. All participants’ data and information will be stored for this research as group data.

### Ethical Considerations

Ethical approval was obtained from the Research Ethics Board at the National University of Malaysia (UKM PPI/111/8/JEP-2021-894; January 28, 2021). Detailed information on the intervention and recruitment process is provided in the next sections.

## Results

This protocol, the informed consent and other relevant documents were reviewed and approved before the conduct of the research. Letters of extension and ethics approval were obtained from the Research Ethics Board at the National University of Malaysia. Amendments will be communicated to investigators, ethics review boards and publishing journals.

By January 2025, a total of 28 participants have been recruited into the study, comprising 14 in the intervention and 14 in the control group. Only 2 participants withdrew from the intervention group mainly due to a change in location and issues relating to data privacy and security. It is expected that participant recruitment will be completed by 30 January 2025. Preliminary analysis is ongoing, and the results suggest improvement in the investigated outcomes among participants in the intervention group across time. Final analysis will be conducted by March 2025, upon completing the data collection.

## Discussion

### Principal Findings

ACEs are relatively common, trigger substantial suffering and are well-documented as a risk factor for diverse physical and mental health conditions throughout life. While primary prevention is pertinent in reducing ACE, the deleterious short and long-term consequences of ACE can be mitigated by selective prevention.

This study adopted the multisystemic approach to resilience “R2,” which recognizes the importance of rugged qualities and access to resources among individuals affected by ACEs. As discussed earlier, the multisystemic model of resilience emphasizes 2 broad aspects, encompassing personal and environmental change [37]. We considered these 2 aspects as pertinent in addressing the consequences of ACEs among young adults in Malaysia, aligning with recommendations from previous studies to approach the problem in a multidimensional manner [8,38]. This study will be the first attempt to adopt an evidence-informed intervention for resilience-building among Malaysians with ACEs. In addition, this research is among the few interventions that explicitly entail both coping strategies and creating better-resourced environments around individuals affected by ACEs.

It is anticipated that the resilience-building intervention will have moderate to strong effects by ameliorating mental health, and stress, and improving participants’ resilience and resource finding compared with those in the control group. These expected findings are consistent with the report by Chandler et al [21], whereby a resilience intervention centered around 4 main components (ie, active coping, building strength, cognitive flexibility, and social support) was effective in mitigating the adverse effects of ACEs.

Our intervention is structured into 5 sessions, focusing on self-exploration and social support, coping techniques and coping skills, resource finding, spirituality, and resilience-building. These components are expected to enhance participants’ internal capacities such as self-regulation and problem-solving, thereby harnessing opportunities to achieve success in life [39]. By educating them on how to identify and gain better access to external resources, they will be motivated to accomplish life tasks and be more optimistic [42]. These events will assist in building resilience, facilitating participants’ interaction with the world around them, and using available resources effectively regardless of being exposed to adversities.

This intervention aims to focus on vital resilience components which are emotion regulation, active coping and goal setting, cognitive flexibility, physical health, mindfulness-based stress reduction, social support, self-exploration, resources and navigation, financial planning, and spirituality and religion. One of the weaknesses of this intervention is that these components will be monitored by informal assessment such as observation, tracking, and recap to ensure participants have learned and acquired the tools and knowledge. It is also difficult to ensure participants from the control group will not receive any form of therapy during the whole duration of the intervention. Hence, the therapist has to check in with the group from time to time to ensure their well-being is taken care of. If needed, the therapist will conduct a post-group counselling session with an individual who is distressed by the intervention. Given that the participants were selected from a pool of individuals obtaining a specific level of ACEs, the findings might not be generalized to the entire population of people with ACEs in the country.

### Conclusion

This is the first full clinical trial study investigating resilience-building intervention for youths with ACEs in Malaysia. There are limited studies evaluating the effectiveness of resilience-building interventions combining mental health and physiological responses as outcome measures in Malaysia. Thus, this study conceptualizes resilience from a biopsychosocial-ecological perspective and adapts resilience-building intervention in the Malaysian context. This study aims to develop resilience-building intervention modules specifically for Malaysia, focusing on the process of adapting the modules and modifications according to the Malaysian culture. This will be one of the first studies to provide evidence in the context of RCTs for resilience-building intervention combining self-report and physiological measures (ie, saliva and heart blood pressure) among individuals with ACEs. The findings will assist relevant authorities in the health and policy sectors to develop effective strategies for addressing the negative impacts of ACEs on the vulnerable population in Malaysia.

## Abbreviations

ACE: adverse childhood experience
ACE-Q: Adverse Childhood Experience Questionnaire
CBT: cognitive behavioral therapy
COPE: Brief Coping Orientation to Problems Experienced Scale
HRB: high-risk behavior
PHQ-9: Patient Health Questionnaire-9
PSS-10: Perceived Stress Scale-10
RCT: randomized controlled trial
SPWB: Ryff’s Scale of Psychological Well-being
WA-PR: Work Ability-Personal Radar
WHOQL-BREF: World Health Organization Quality of Life Assessment

## Data Availability and Dissemination Policy

Group data will be available on the Australia New Zealand Clinical Trials Registry. The public will have access to full protocol and group-level datasets. The investigator will publish trial results to participants and health care professionals through publication and data-sharing arrangements on the Australia New Zealand Clinical Trials Registry. The public will have access to the full protocol. Data will be available upon the study’s completion.
